# Regression of Cardiac Lymphoma With Chemotherapy

**DOI:** 10.1016/j.jaccas.2023.102166

**Published:** 2023-12-17

**Authors:** Juan Fernández-Martínez, Martín Descalzo-Buey, Irene Menduiña-Gallego, Sandra Pujadas-Olano, David Viladés-Medel, Mario Salido-Iniesta, Diego A. López-Mora, Silvana Novelli-Canales, Ruth J. Orellana-Fernández, Rubén Leta-Petracca

**Affiliations:** a Department of Cardiology, Hospital de la Santa Creu i Sant Pau, Barcelona, Spain; b Department of Nuclear Medicine, Hospital de la Santa Creu i Sant Pau, Barcelona, Spain; c Department of Hematology, Hospital de Sant Creu i Sant Pau, Barcelona, Spain; d Department of Pathology, Hospital de Sant Creu i Sant Pau, Barcelona, Spain

**Keywords:** cardiac lymphoma, cardiac magnetic resonance, myocardial inflammation, positron emission tomography

## Abstract

A patient was admitted for chest pain with electrocardiographic changes, and cardiac magnetic resonance showed focal myocardial hypertrophy secondary to edema. Combined positron emission tomography and computed tomography corroborated foci of myocardial hypermetabolism, as well as multiple adenopathies consistent with lymphoma in the biopsy. Hypertrophy and edema regressed with chemotherapy.

## Graphical abstract

**Figure:**
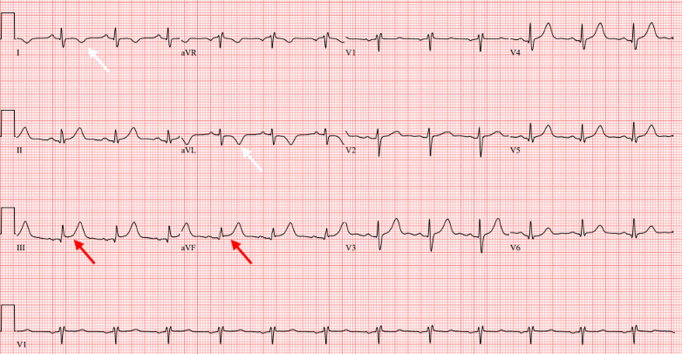

## History of Presentation

**Figure 1:**
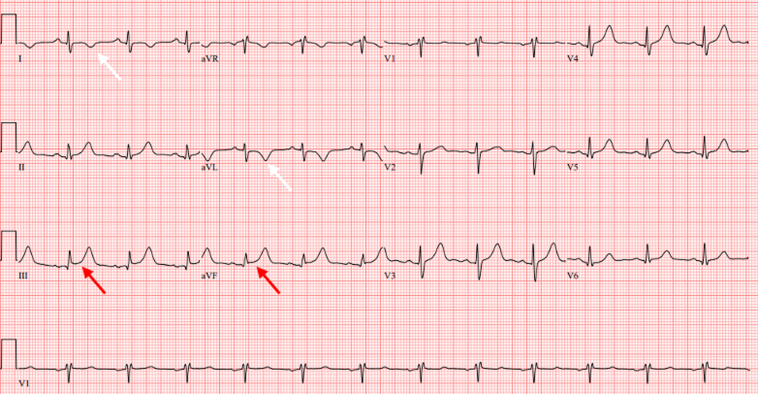
Electrocardiogram on Arrival The tracing shows slight elevation of the ST-segment in the inferior leads (red arrows) and a negative T-wave in leads I and aVL (white arrows).

A 49-year-old woman from Peru presented to the emergency department with atypical but intense chest pain with no clear relationship with physical exertion from 5 days earlier. On arrival, she was asymptomatic, her heart rate was 75 beats/min, her blood pressure 113/65 mm Hg, her respiratory rate was 18 breaths/min, and her oxygen saturation was 98%. The physical examination was normal, but the electrocardiogram (ECG) (Figure 1) showed a slight elevation of the ST-segment in the inferior leads and depression of the ST-segment and a negative T-wave in leads I and aVL, without evolutionary changes in the successive ECGs. On the initial laboratory tests, she showed a high-sensitivity cardiac troponin T value of 18 ng/L (normal value [NV] <13 ng/L) with no other significant findings.

**Learning Objectives**

• To be able to make a differential diagnosis of myocardial injury with multimodality imaging.
• To keep in mind extracardiac causes of myocardial injury.
• To correlate the different imaging modalities and their usefulness.
• To keep trying to establish a definite and accurate diagnosis.

An initial bedside echocardiogram (Videos 1, 2 and 3) showed preserved biventricular ejection fraction, although mild focal hypokinesia and thickening of the basal anterolateral segment were detected.

## Past Medical History

The patient had no previous medical history or cardiovascular risk factors. However, she reported a previous history of 3 months of limb polyarthralgias and migratory dysesthesias.

## Differential Diagnosis

**Figure 2:**
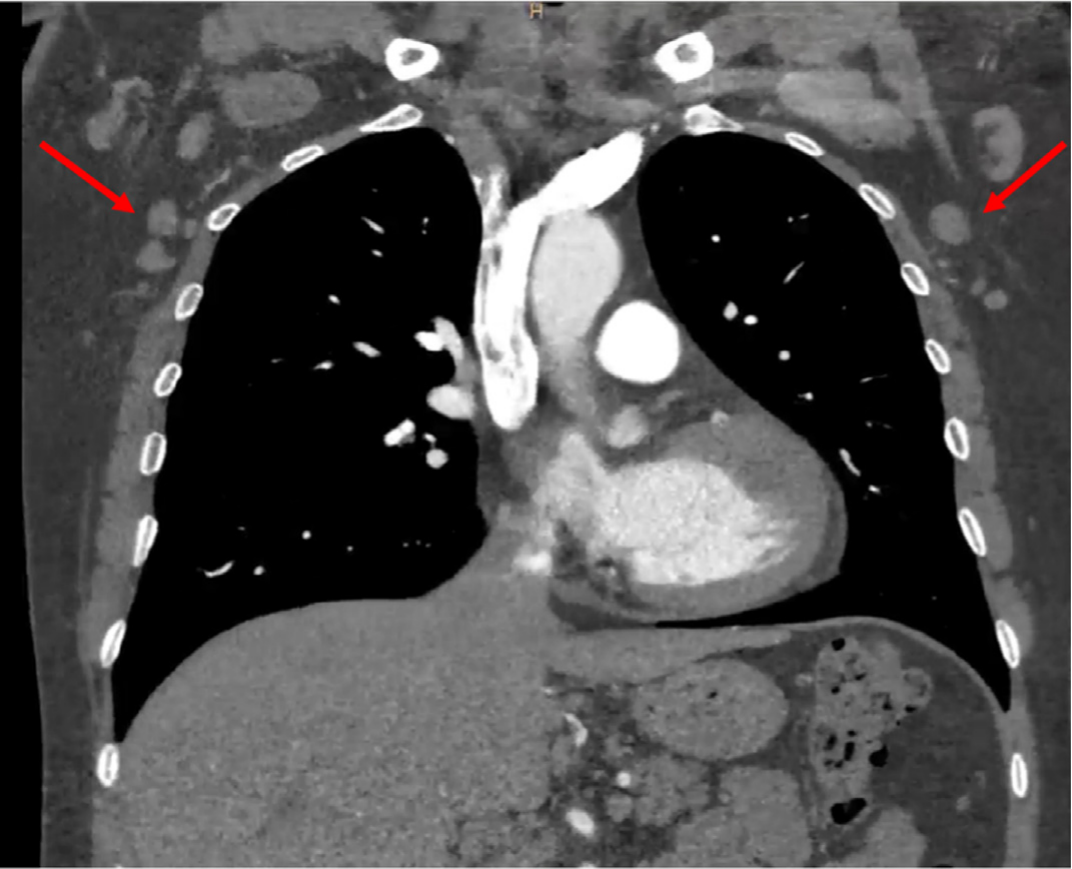
Computed Tomography Scan The image shows several small axillary and mediastinal adenopathies (arrows).

Given the recurrence of chest pain with a slightly elevated troponin level and the ECG findings, ST-segment elevation myocardial infarction was the primary working diagnosis. Therefore, emergency invasive coronary angiography was performed, and it showed no significant coronary artery disease. A thoracic computed tomography (CT) scan (Figure 2) was also performed to rule out other life-threatening causes of intense chest pain, such as acute aortic syndrome or pulmonary embolism, and it revealed several small axillar and mediastinal adenopathies**.** The patient was admitted to the cardiology ward.

## Investigations

**Figure 3:**
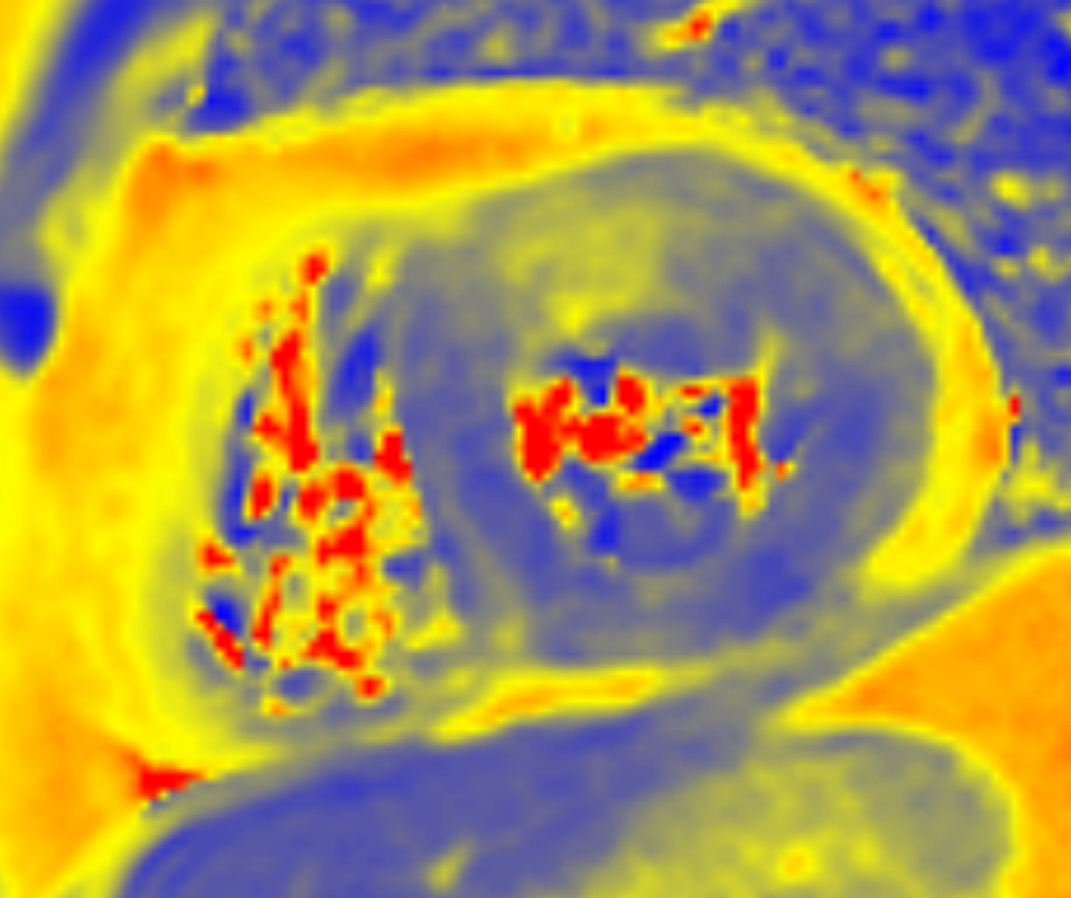
T2 Mapping on the Initial Cardiac Magnetic Resonance The image shows focal edema on the anterior segment (blue, values <55 ms; yellow, values >55 ms).

**Figure 4:**
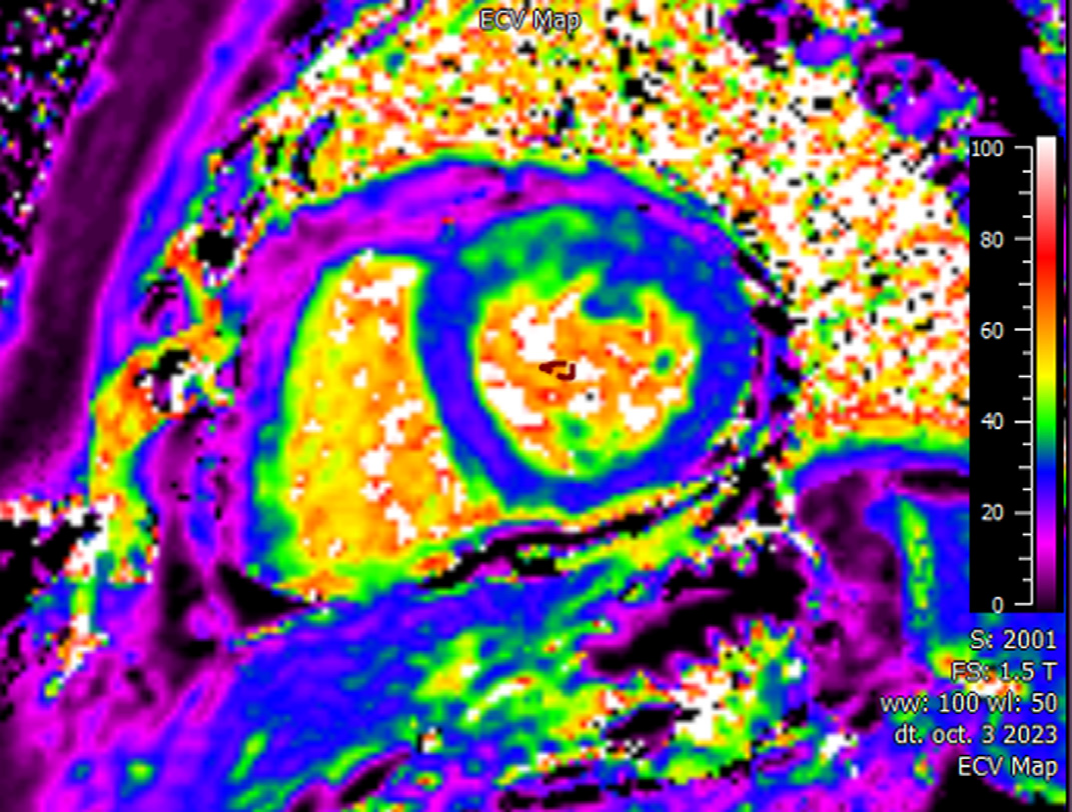
Extracellular Volume Map on the Initial Cardiac Magnetic Resonance The imaging shows high extracellular volume on the anterior segment (blue, normal extracellular volume; green, high extracellular volume).

**Figure 5:**
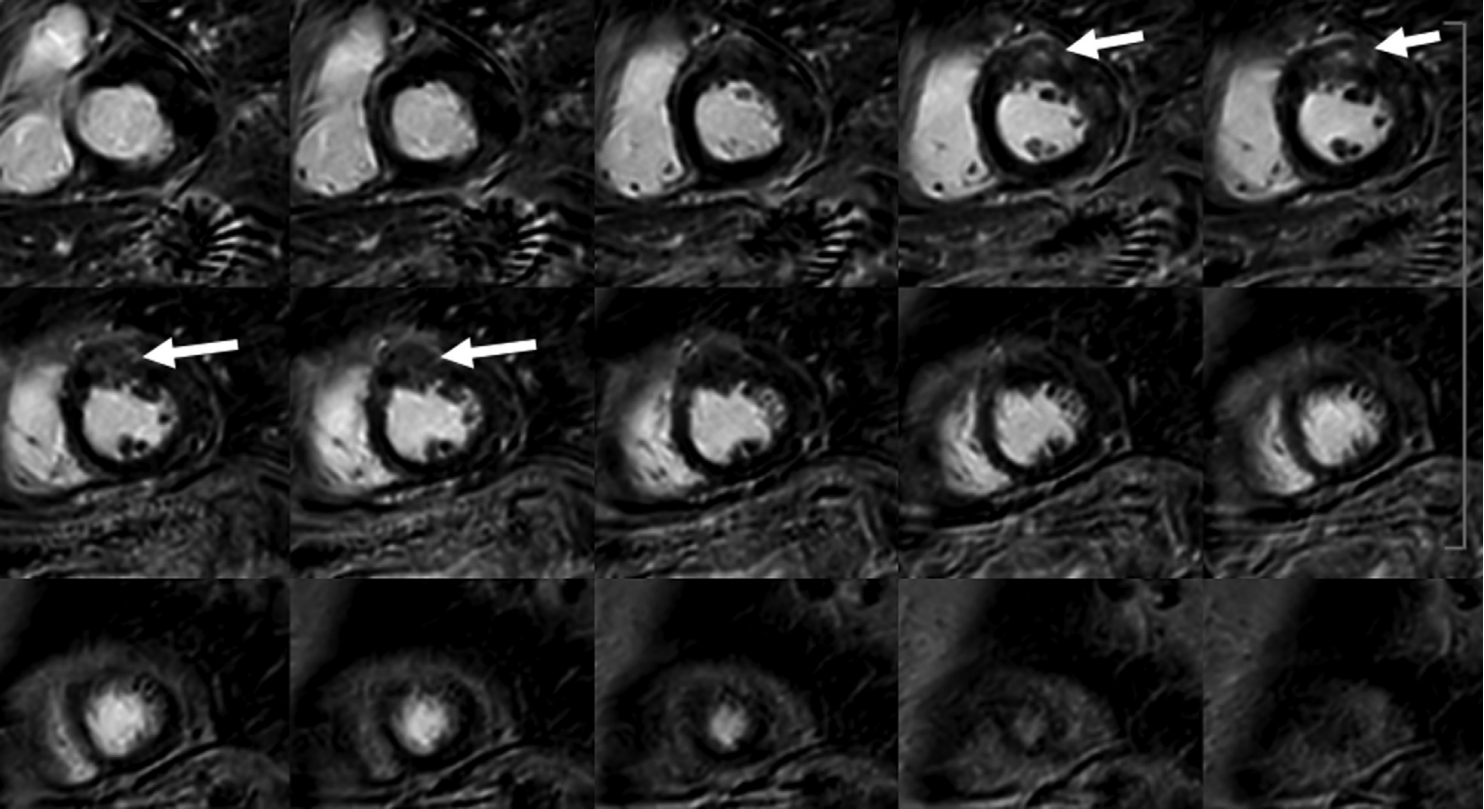
Late Gadolinium Enhancement Sequences on the Initial Cardiac Magnetic Resonance The imaging reveals an intramyocardial heterogeneous and faint contrast medium retention in the hypertrophic segments (arrows).

Initial cardiac magnetic resonance (CMR) was performed, with a 1.5-T scan showing a nondilated left ventricle with preserved ejection fraction but with significant thickening of the basal and midanterior and anterolateral segments (maximal 17 mm) (Video 4). Those segments showed high T2 (>65 ms; NV <55 ms) and T1 (1,230 ms; NV <1,050 ms) mapping values (Figures 3 and 4), with an increased extracellular volume of 33% (NV<30%), suggesting the presence of abundant myocardial edema. Late gadolinium enhancement sequences revealed an intramyocardial heterogeneous and faint contrast medium retention in the same hypertrophic segments (Figure 5). The report concluded with a probable diagnosis of myocardial pseudohypertrophy secondary to important myocardial inflammation of unknown origin.

Because myocarditis was suspected, anti-inflammatory treatment was started, and the patient achieved complete remission of chest pain. Laboratory tests were amplified with viral serologic studies (SARS-CoV-2, Epstein-Barr, and parvovirus B19) and a complete rheumatologic profile, all of which had negative results. The proteinogram revealed an isolated elevation of β_2_-microglobulin of 3.38 mg/L (NV <1.8 mg/L).

**Figure 6:**
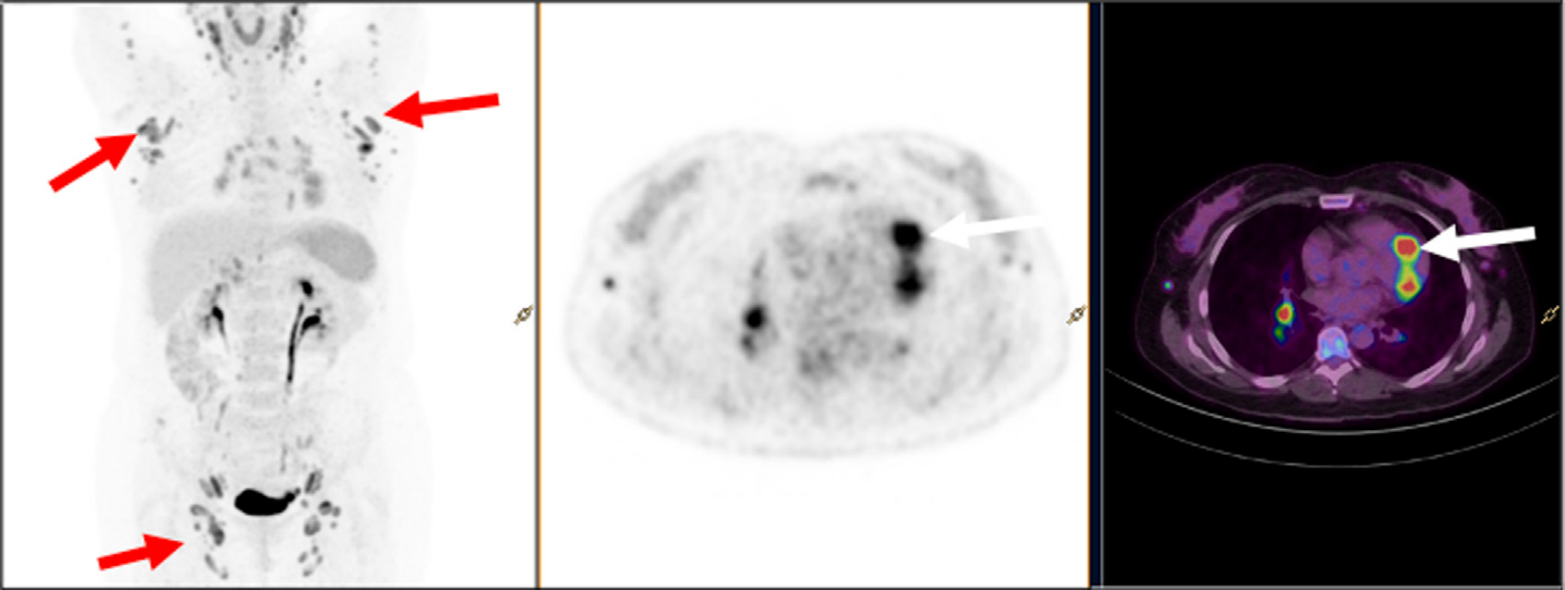
Initial Fluorine-18 Fluorodeoxyglucose Positron Emission Tomography/Computed Tomography The scan shows polyadenopathies (red arrows) and myocardial hypermetabolic foci (white arrows).

The presence of adenopathies on the thoracic CT scan raised the suspicion of possible underlying cardiac inflammatory disease. Therefore, the patient underwent a positron emission tomography (PET)/CT scan with fluorine-18 fluorodeoxyglucose (FDG). The scan revealed multiple supradiaphragmatic and infradiaphragmatic hypermetabolic lymph nodes with splenic and subcutaneous infiltration, suggesting an active lymphoproliferative process. Myocardial hypermetabolic foci, matching the inflamed segments noted on CMR (Figure 6), were also observed.

**Figure 7:**
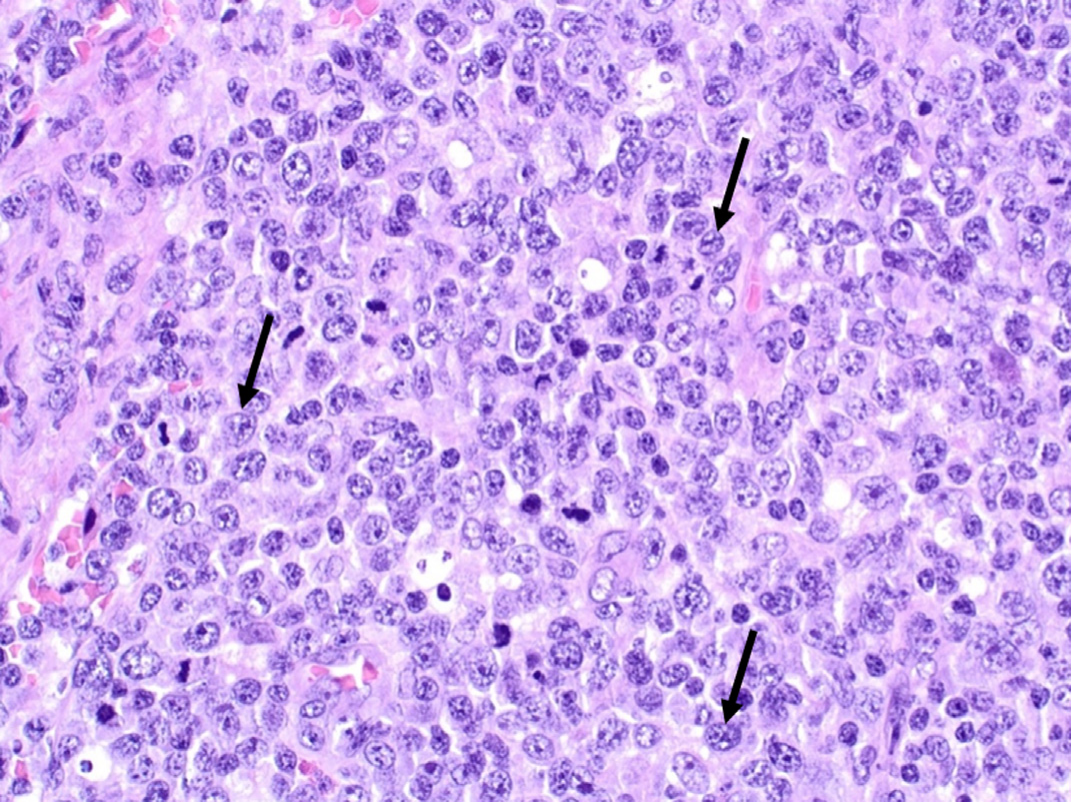
Biopsy of an Inguinal Node The biopsy shows proliferation of lymphocytes with a large nucleus compatible with large T-cell lymphoma seen on hematoxylin and eosin stain (arrows) (original magnification, ×100).

The diagnostic work-up was completed with a biopsy of inguinal adenopathy that showed neoplastic proliferation made up of lymphocytes with a large nucleus compatible with large T-cell lymphoma (Figure 7), CD30 positive and anaplastic lymphoma kinase negative. A bone marrow biopsy was also obtained from the iliac crest, and it showed focal centromedullary infiltration resulting from T-lymphoproliferative syndrome (CD30, CD2, CD5 positive) with preserved hematopoiesis.

## Management

Chemotherapy was started with the cyclophosphamide, doxorubicin, vincristine, and prednisone regimen, and the patient completed 4 cycles. During follow-up, she remained asymptomatic for cardiovascular symptoms, with normalization of the ECG. Unfortunately, she presented with progression of the lymphoma with infiltration of the central nervous system. Because the patient came from an endemic region, serologic testing for human T-cell lymphotropic virus type 1 was conducted, and the result was positive. Therefore, T-cell leukemia/lymphoma was concluded as the final diagnosis, which has a much worse prognosis, and treatment was switched to an etoposide, methylprednisolone, cytarabine, and cisplatin–type chemotherapy regimen.

## Discussion

Myocardial inflammation as a cause of chest pain is relatively common. CMR is a cornerstone cardiac imaging technique to assess the myocardial injury because of the modality’s high sensitivity and accuracy in the characterization of thickness, edema, or fibrosis. In this regard, CRM and PET/CT are well established in position papers and clinical guidelines in this clinical setting.^1^

Conversely, myocardial inflammation as the first manifestation of a lymphoproliferative process is described in some case reports, usually associated with ventricular dysfunction and its complications, such as arrhythmias^2^ or heart failure^3^; chest pain is not a common first presentation. Secondary cardiac involvement from lymphoma is a relatively frequent occurrence, reported in up to 25% of patients^4^; this incidence is probably underestimated because of a high percentage of asymptomatic cases. Myocardial involvement of adult T-cell leukemia/lymphoma is often detected during autopsy; however, the development of cardiac symptoms is extremely rare, with only 7 registered cases.^5^

Other presentations of lymphoproliferative processes in the heart are primary tumors, where lymphomas represent just 10% of primary malignant heart tumors and approximately 1% of all primary cardiac tumors. CMR also has a key role in this entity. Primary cardiac lymphomas more frequently involve the right side of the heart and usually manifest with right-sided congestive heart failure.^6^

In our patient, acute chest pain was the first guiding symptom that led to the final (and relatively early) diagnosis. Unfortunately, given the severity of the hematologic disease, her prognosis was eventually equally poor.

## Follow-Up

**Figure 8:**
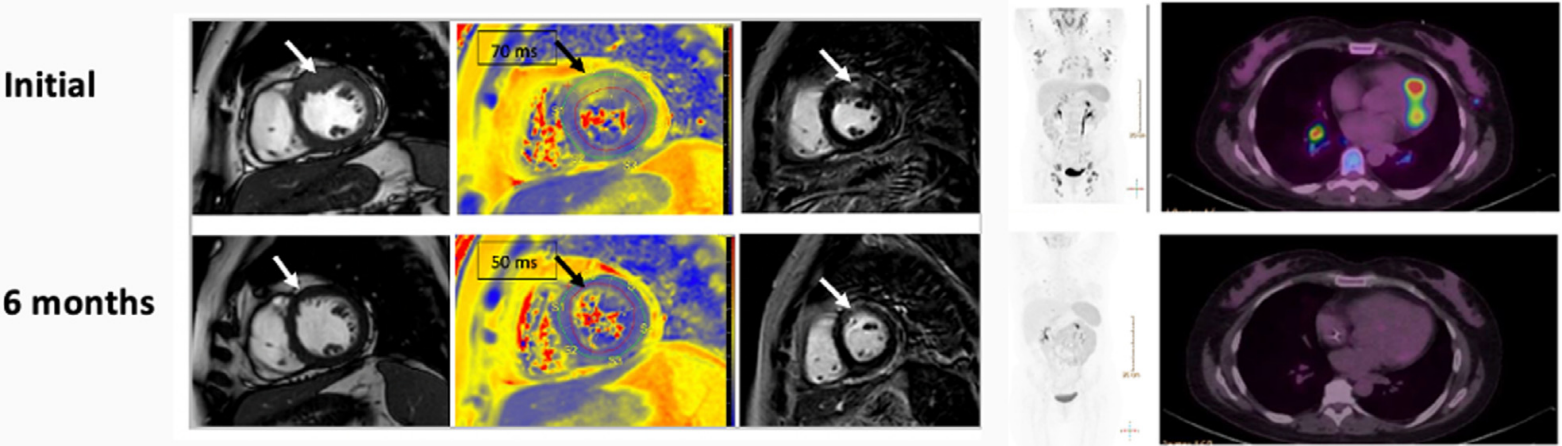
Comparison of Initial and Follow-Up Imaging (Top left) Initial cardiac magnetic resonance (from left to right: cine, T2 mapping, and late gadolinium enhancement sequences) and (top right) initial fluorine-18 fluorodeoxyglucose positron emission tomography/computed tomography scans. (Bottom left) Follow-up cardiac magnetic resonance and (bottom right) follow-up fluorine-18 fluorodeoxyglucose positron emission tomography/computed tomography scans. The arrows point out the inflamed segment that heals with chemotherapy.

Follow-up CMR was performed, and it showed preserved biventricular function with resolution of myocardial edema (normalization of the T1 and T2 mapping values) and regression to normal wall thickness, leaving residual soft myocardial fibrosis in the previously inflamed segments (Figure 8). The subsequent FDG PET/CT scan confirmed the absence of uptake at the myocardial level despite systemic progression of the lymphoproliferative process (Figure 8). Finally, a third line of chemotherapy was started without neurological improvement. Despite an excellent documented cardiac response, she presented with refractory cranial hypertension, which led to a fatal outcome 9 months after the initial diagnosis.

## Conclusions

Myocardial injury is usually a diagnostic challenge that is not always possible to resolve. The advancement of different multimodal imaging techniques allows us to obtain more information, leading to increasingly precise and complete diagnoses. It is a difficult scenario, and often the cause is not primarily cardiac, so it is important to keep an open and objective mind.

## Funding Support and Author Disclosures

The authors have reported that they have no relationships relevant to the contents of this paper to disclose.

## Appendix

Video 1 Parasternal Long-Axis View on the Initial Echocardiogram

Video 2 Parasternal Short-Axis View on the Initial Echocardiogram

Video 3 Apical 5-Chamber View on the Initial Echocardiogram

Video 4 Initial CMR Showing Anterior and Anterolateral Wall Thickening
